# Dynamics of HIV reservoirs following repeated anti-SARS-CoV-2 vaccination

**DOI:** 10.3389/fimmu.2026.1829485

**Published:** 2026-05-29

**Authors:** Jessica Lu, Ali Ahmad, Nicolas Chomont, Cecilia T. Costiniuk

**Affiliations:** 1 Faculty of Medicine and Health Sciences, McGill University, Montreal, QC, Canada; 2 Department of Microbiology, Immunology & Infectious Diseases, Centre Hospitalier Universitaire (CHU) Sainte-Justine Research Center, University of Montreal, Montreal, QC, Canada; 3 Département de Microbiologie, Infectiologie et Immunologie, Université de Montréal, Montreal, QC, Canada; 4 Centre de Recherche du Centre Hospitalier de l’Université de Montréal (CHUM), Montreal, QC, Canada; 5 Infectious Diseases and Immunity in Global Health, Research Institute of McGill University Health Centre, Montreal, QC, Canada; 6 Department of Medicine, Division of Infectious Diseases and Chronic Viral Illness Services, McGill University Health Centre, Montreal, QC, Canada

**Keywords:** HIV, HIV reservoirs, inflammation, PWH, SARS-CoV-2, vaccination

## Abstract

Due to persistent immune dysfunction, People with HIV (PWH) are at increased risk of more severe infections with SARS-CoV-2 and hospitalizations. In several countries, they were listed as a priority group for receiving anti-SARS-CoV-2 vaccines when these vaccines were in scarce supply. These prophylactic vaccines do not completely prevent infections in vaccinated individuals, especially with a heterologous vaccine strain. However, they persistently reduce the severity of breakthrough infections, hospitalization rates and deaths. Although antiretroviral therapy (ART) suppresses HIV replication below the levels detectable by the assays used routinely in clinical care, it does not eliminate viral reservoirs; a small pool of infected cells carrying HIV proviruses integrated into the genomes of CD4^+^ T cells and a subset of myeloid cells. Since vaccines work by stimulating these cells, they may theoretically reactivate latent HIV, increase plasma viremia and modulate the size of the HIV reservoir. The apprehension of HIV reactivation in PWH on ART prompted several studies in the past five years to investigate the impact of anti-SARS-CoV-2 vaccination on the markers of HIV persistence. Collectively, these studies have shown that the vaccination is generally safe in PWH on ART and induces only transient increases in viremia with no measurable impact on the size of the reservoir. However, in PWH with unsuppressed viremia, the vaccination was accompanied by more prolonged increases in viremia and in the frequencies of HIV-infected cells. In this review, we discuss these studies, their outcomes, their implications for HIV cure and propose topics for further research.

## 1 Introduction

Since the emergence of the COVID-19 pandemic, declared in 2020, several studies have demonstrated that people with HIV (PWH) often experience a higher rate of adverse outcomes, more severe disease and greater rates of hospitalizations compared to individuals without HIV (PWOH) (1–3). Rather than being solely the result of an HIV diagnosis, more severe COVID-19 outcomes are associated with comorbidities, low CD4^+^ T-cell counts, and detectable viral load—either due to the absence of ART and possibly due to incomplete viral suppression despite antiretroviral therapy (ART) (1–3). As such, in some countries, PWH—typically those with advanced disease or other comorbidities—were considered as a priority group for vaccination when anti-SARS-CoV-2 vaccines became available and were scarce. The effectiveness of these prophylactic vaccines in preventing infections is variable and transient, as evidenced by breakthrough infections in the vaccinated individuals. However, they are persistently effective in reducing the severity of clinical symptoms, hospitalizations and deaths in the vaccinated individuals. Consequently, breakthrough infections result in milder, often asymptomatic cases in the vaccinated individuals compared with those in non-vaccinated individuals. Since their rollout in December 2020, SARS-CoV-2 vaccines have averted millions of deaths in the world that might have been caused by this novel coronavirus (4). Furthermore, the vaccine-induced immunity is not durable and wanes over time, requiring booster doses (5).

Our team and others have demonstrated favorable safety and tolerability profiles for anti-SARS-CoV-2 vaccines in PWH (6, 7). However, PWH tend to lack confidence in the vaccines—especially in certain subpopulations of PWH—compared with PWOH and have lower vaccine uptake (8). Our previous work has shown that the reasons for the lack of confidence are multifactorial (9). One of the initial reasons for lower vaccine confidence in PWH versus the general population was uncertainty about their effectiveness and perceived fear of HIV rebound, especially given the robust immune responses induced by mRNA-based vaccines. The concern of HIV rebound was not unfounded, as other vaccines, including those against influenza, Hepatitis B virus (HBV) and pneumococcus, can induce HIV reactivation, leading to detectable increases in plasma HIV RNA levels in PWH (10–14). However, in most cases, increases in viremia were transient (viral blips), of low magnitude and without any significant change in HIV reservoir size. Nevertheless, a clinical trial of Twinrix^®^ (a vaccine from GlaxoSmithKline for HBV and HAV) in HIV-positive children on ART observed a significant increase in central memory CD8^+^ T cells and a decrease in activated CD4^+^ T cells, accompanied by increased HIV RNA. Importantly, the authors also documented a significant decrease in HIV reservoir size (15). A similar decrease in the reservoir size was also reported in HIV-positive pregnant women on ART after influenza vaccination (14, 16).

The most widely used SARS-CoV-2 vaccines are mRNA vaccines, which are based on nucleotide-modified mRNA which encodes the Spike glycoprotein (S) of SARS-CoV-2, the causative agent of COVID-19. They activate the immune system of the vaccinees by different mechanisms discussed in the following text. The mRNA component of these vaccines activates the innate immune system at cellular and intracellular levels (17). The S-encoding mRNA in these vaccines is delivered in lipid nanoparticles (LPN). The ionizable component of the LPN also acts as an adjuvant, stimulating immune cells (17). This immune stimulation may potentially activate HIV in latently infected cells in PWH. Furthermore, *in vitro* studies have shown that S binds its cellular receptor, Angiotensin Converting Enzyme (ACE)-2, and reactivates HIV in latently infected cells through activation of the mTOR-S6K-NF-κB pathway (18–21). A recent publication, though not peer-reviewed, also demonstrated that the S protein mediates activation of HIV transcription in a T cell line model of latent HIV infection by activating mTOR (20). This observation is important given that the S protein can be detected in the circulation of vaccinated individuals for at least several weeks following immunization (22). Furthermore, Pfizer/BioNTech’s BNT162b2 mRNA vaccine induces HIV reactivation when incubated ex vivo with PBMC from SARS-CoV-2-naïve PWH who are on ART with undetectable viremia (23). The reactivation was mediated by activation of the RIG-1-, TLR7/8-, and TNF-induced signaling pathways (23). The viral reactivation and inflammatory mediators lead to further *de novo* infection of activated naïve CD4^+^ T cells as well as expansion of latently infected CD4^+^ T cells. The viral reactivation, however, will also expand HIV-specific CD8^+^ T cells resulting in the elimination of productively HIV-infected CD4^+^ T cells. Together these mechanisms, induced by the vaccines, will affect HIV reservoirs in the vaccinated PWH. The studies (20, 21, 23) highlight the potential of the anti-SARS-CoV-2 vaccines, analogous to latency reversing agents, to reactivate latent HIV, stimulate HIV-specific immunity and modulate viral load and reservoir size in the vaccinated PWH on ART.

## 2 SARS-CoV-2 vaccine landscape

Globally, vaccine strategies for SARS-CoV-2 varied considerably, with a key distinction being the types of vaccines deployed across regions. mRNA-based vaccines included Pfizer-BioNTech’s BNT162b2/Comirnaty and Moderna’s mRNA-1273/Spikevac (24). These vaccines were developed within one year following the emergence of the COVID-19 pandemic. They are based on nucleotide modification, replacing uridine in mRNA with 1’-methylpseudouridine, to increase stability and avoid innate immune sensing of the mRNA. As mentioned above, the mRNA in these vaccines encodes the Spike glycoprotein (S) of SARS CoV-2, and is delivered in LNP. These vaccines were predominantly used in North America, Western Europe, and several high-income countries in other regions, such as Japan and Australia (25, 26). In contrast, many low- and middle-income countries in Africa, South America, the Middle East, and parts of Asia relied more on non-mRNA-based vaccines, such as adenoviral vector-based vaccines that deliver DNA of the S protein (e.g., AstraZeneca’s ChAdOx1, Johnson & Johnson’s Ad26.COV2.S), inactivated whole virus vaccines (e.g., Sinopharm’s BBIBP-CorVac, Sinovac’s CoronaVac, Bharat Biotech’s Covaxin), and protein subunit vaccines (e.g. Anhui Zhifei Longco’s Zifivax or ZF2001) (24–26). In ChAdOx1 and Ad26.COV2.S, the S protein encoding DNA is vectored in modified chimpanzee’s adenovirus (ChAd) and modified human adenovirus type 26 (Ad26), respectively. The modifications include deleting the E1 and E3 regions from the adenoviral genomes, rendering the adenoviruses replication-incompetent and safe. The deletions also provide room for the insertion of DNA for the target antigen (S). Human populations have lower levels of immunity against Ad26 compared to Ad5. The pre-existing immunity may lead to undesired consequences, as was demonstrated for an Ad5-vectored HIV vaccine that increased the risk of HIV acquisition compared with unvaccinated controls (27). Inactivated vaccines are, in fact, whole intact SARS-CoV-2 killed with heat, radiation or chemicals such as β-propiolactone. The subunit vaccine ZF2001 uses two copies of the receptor-binding domain (RBD) of the S protein joined in tandem as an antigen (28). SARS-CoV-2 neutralizing antibodies mainly target RBD, block the RBD-ACE-2 interaction and prevent viral infection. As anti-SARS-CoV-2 vaccines are highly heterogeneous regarding their construction, antigens, delivery systems and adjuvants, the types of these vaccines must be identified when comparing and interpreting vaccination outcomes.

The mRNA-based vaccines show a higher efficacy rate (>90%) in preventing infection with the vaccine strains of the virus compared with inactivated vaccines (50-79%); however, both are highly effective in preventing severe disease from the infection (29, 30).

## 3 HIV reservoirs and their measurements

Cellular HIV reservoirs refer to a population of cells present in the body of a PWH that harbor transcriptionally silent (partially or completely) and replication-competent HIV DNA (proviruses) integrated in their genomes (31–35). ART suppresses HIV replication by preventing infection of new target cells but does not eliminate integrated proviruses. The viral reservoirs are the main hurdle in achieving an HIV cure, as the virus rebounds within weeks upon ART cessation. During an untreated infection, HIV DNA exists both in integrated and unintegrated (episomal and linear) forms. The latter decays over time when HIV replication is suppressed by ART. Thus, in PWH on ART with suppressed viral replication, most HIV genomes are integrated within the chromatin of the infected cells (36, 37). Studies have shown that more than 90% of the integrated proviruses persisting in PWH on ART are genetically defective, meaning that they are incapable of replicating and causing viral rebound upon ART cessation (38–40). However, they have been associated with chronic inflammation, immune activation and comorbidities, as their activation may yield viral transcripts and proteins or, in rare cases, even non-infectious virions (41–44). The size of the latent HIV reservoir has been historically measured by the quantitative viral outgrowth assay (QVOA), which provides a minimal estimate of the frequencies of cells harboring replication-competent HIV genomes. In this limiting dilution assay, frequencies of resting CD4^+^ T cells harboring mitogen-inducible and replication-competent proviruses are quantified after their co-culture with HIV-susceptible cells (45, 46). A drawback of the QVOA is that only a fraction of latently infected cells are induced by mitogens, which may underestimate the size of the HIV reservoir (38). Furthermore, the assay is cumbersome and time-consuming.

A convenient estimate of the size of the HIV reservoir can be obtained by measuring the frequencies of cells harboring HIV DNA (either total or integrated) by qPCR on DNA extracted from PBMC or CD4^+^ T cells (47, 48). These measures, however, do not discriminate between intact and defective proviruses, and therefore largely overestimate the size of the HIV reservoir (49). To discriminate between and quantify intact and defective proviral genomes, the Intact Proviral DNA Assay (IPDA) was recently developed (50). It is based on the amplification of two conserved regions of the HIV genome, which are frequently mutated in defective proviruses, i.e., the packaging signal (Ψ) and the rev response element (RRE). Intact viral genomes are positive for both of these targeted regions. IPDA provides a rapid and convenient measure of the genetically intact HIV reservoir. However, it misclassifies some genomes and, more importantly, does not measure inducibility. Indeed, it is now well-described that a fraction of intact provirus may not be induced if they are integrated in a transcriptionally silent region of the cellular genome (51, 52). To address this issue, measures of the “functional” or inducible reservoir have been developed. The measure of cell-associated viral HIV RNA (CA-RNA) reflects the magnitude of the transcriptionally active viral reservoirs (53). It is also used to measure the effectiveness of latency reversing agents, i.e., the agents that can reactivate and induce transcription from latent proviruses (54).

Another assay, used in some studies, to estimate the size of the HIV reservoir is the Tat/Rev-Inducible Limiting Dilution Assay (TILDA) (55). This method measures the frequency of CD4^+^ T cells that can produce multiply spliced cell-associated viral RNA following stimulation. The assay involves the stimulation of CD4^+^ T cells plated in limiting dilution with PMA/ionomycin, and the detection of Tat/Rev transcripts by nested PCR (55). TILDA can detect ∼1 cell harboring replication-competent HIV provirus per million CD4^+^ T cells.

## 4 Impact of repeated anti-SARS-CoV-2 vaccination on HIV reservoir

The impact of repeated anti-SARS-CoV-2 vaccination on the dynamics of the HIV reservoir has been examined by several groups. These studies are listed and summarized in **Table 1**.

**Table 1:** Studies investigating dynamics of HIV reservoir following anti-SARS-CoV-2 vaccination in PWH.

Article county	Participants’ demography	Recruitment period & Inclusion and Exclusion criteria	Vaccine studied	Reservoir measures & assays	Immune measures/markers examined*	Timing	Key study outcomes	Reservoir finding
Duncan, M. et al. (56) Canada	N = 62 Vaccination: 43 (69%) BNT162b2 + BNT162b2 16 (26%) mRNA-1273 + mRNA-1273 3 (4.8%) BNT162b2 + mRNA-1273 Median Age (years): 43 Sex 55 (89%) male 7 (11%) female Median time on ART: 6 years Baseline CD4^+^ T cell count per µl: 725 (IQR 475, 915) Viral load (HIV RNA copies/ml) 51 (82%) pVL < 20	Inclusion criteria: -HIV diagnosis -Receiving ART Exclusion criteria: Not specified Recruited: December 2020-August 2022	Moderna or Pfizer-BioNTech mRNA-based SARS-CoV-2 vaccines	HIV reservoirs – IPDA Plasma HIV RNA levels – cobas Ampliprep/Taqman HIV-1 Test v2.0 or Cobas HIV-1 Test on Cobas 6800	Vaccine-induced anti-SARS-CoV-2 Spike antibodies in serum – Roche Elecsys Anti-S assay	(1) Pre-vaccination (2) 1 month after first dose (3) 2 months after second dose	Cohort analysis: - Plasma viremia post-vaccination - Reservoir size post-vaccination Population analysis: -Frequency of detectable HIV test results	Intact HIV copies not significantly changed between baseline and either post-vaccine visit. 5’-defective, 3’-defective, and total proviral burdens not changed significantly between baseline and either post-vaccine visit.
Stevenson EM et al. (23) USA	n = 35 ART-treated adults with undetectable viral load (1) New York cohort (n=20) (2) Vancouver cohort (n=15 from the cohort of Duncan et al. (56)	Inclusion criteria: -ART treatment -Undetectable viral load -18–89 years of age -Sustained HIV suppression for at least 1 year -HIV viral load <50 copies/mL -mRNA vaccination Exclusion criteria: -Contraindication for SARS-CoV-2 vaccination -Plasma HIV RNA >200 copies/mL -Known anemia with hemoglobin <10gm/dL -Discontinuation of ART for 7 or more consecutive days	Moderna or Pfizer-BioNTech mRNA-based SARS-CoV-2 vaccines	HIV RNA – qPCR HIV reservoirs – IPDA; TILDA (n=4)	T-cell responses – gzm-B ELISPOT, flow cytometric analysis of CD69 expression as AIM assays	qPCR measurement 48 h following ex vivo treatment of PBMCs from participants *In vivo* HIV reactivation: (1) baseline (up to 6 weeks before vaccination) (2) Approx. 2 weeks after first dose (3) Approx. 2 weeks after second dose	HIV reactivation post-vaccination	SARS-CoV-2 mRNA vaccination induced modest and transient transcriptional activation of latent HIV in individuals on ART, leading to exposure of HIV-infected cells to Nef-specific CD8^+^ T-cell responses. Mechanistically, the BNT162b2 mRNA vaccine activates the RIG-I/TLR–TNF–NFκB axis, which increases HIV transcription ex vivo with minimal global T-cell activation.
DiGirolamo, L. et al. (57) Italy	Woman, 65years old, of European ancestry; Dx HIV-1/HCV more than 25 years ago Since then, her HIV-RNA has remained undetectable while she has never received ART	N/A	3 doses of Pfizer-BioNTech mRNA-based SARS-CoV-2 vaccine	HIV-DNA – HIV-1 DNA Test PRO	S-specific IgG; CD4+ T and CD8+ T cell counts; CD4/CD8 T cell ratio	First dose: June 24^th^, 2021 Second dose: July 25^th^, 2021 Third dose: October 2021	N/A	Rebound of plasma HIV-RNA after each dose. Spontaneous reduction of HIV-RNA and total HIV-DNA in peripheral mononuclear cells without ART was observed 10 months after the third dose
Peng, X. et al. (58) China	n = 15 Median Age 32 (IQR 27, 41) Sex 15 (100%) male Median CD4^+^ T count 415/uL (IQR 257.5, 606.5) Viral load All <20 copies/mL	Recruitment period: April 2021 to February 2022 Inclusion criteria: -Males -Aged between 18-65 -HIV diagnosis -At least 1 year of ART -Willingness to receive at least 2 doses of CoronaVac Exclusion criteria: -Severe chronic diseases or acute conditions	At least 2 doses of CoronaVac (an inac-tivated vaccine)	CA-HIV-RNA – HIV-1 RT-PCR (Assay V2, Qiagen) HIV DNA – SUPBIO Quantitative Detection Kit HIV VL – Abbott Real-time HIV-1 assay	T-cell receptor repertoire profiles, clustering and annotation – GLIPH2 Clonal breadth and depth of S-specific T-cells – Compare TRBV gene segments to known S-specific clonotypes in the VDJdb database	Before vaccination, 2 and12 weeks after second dose 2 weeks after third dose	HIV reservoir dynamics, T cell immune response	Transient increase in CA-HIV RNA and HIV DNA after CoronaVac vaccination
Matveev, V (59). Canada	N = 91: 68 PWH and 23 HIV-negative participants Median Age HIV-: 62.0 (IQR 58, 70) HIV+:63.0 (IQR 58, 69) Sex 90 (89%) male 1 (11%) female Race 79 (86.8%) Caucasian; 4 (4.4%) Latino; 2 (2.2%) Black; 2 (2.2%) Middle Eastern; 2 (2.2%) South Asian; 1 (1.1%) East Asian; 1 (1.1% Native Brazilian HIV^+^: PWH Profile 42 immunological responders; 20 immunological non-responders; 5 low-level viremia; 1 LNTP HIV^+^: CD4^+^ T cell count 527 (IQR 364, 665)	Inclusion criteria: ->55 years old -PWH on ART Exclusion criteria: Not specified	D1/D2: -mRNA/mR NA -ChAdOx1/ ChAd26Ox1 -ChAdOx1/ mRNA D3: Moderna or Pfizer-BioNTech mRNA-based SARS-CoV-2 vaccines	HIV DNA – IPDA	Total IgG Ab levels – ELISA assay B-cell frequency – spectral flow cytometry T-cell cytokine responses – dual-color ELISpot	Baseline (V1): up to 24h before first vaccine dose; V2: 10 days post D1; V3: 20 days post D1; V4: 28 days post D1; V4a: up to 3 days before D2; V5: 1 week after D2 V6: 2 weeks post D2; V7: 4 weeks post D2; V8: 24 weeks post D1; V8a: 3 days before D3; 8b: 4 weeks post D3; V8c: 16 weeks after D3; V9: 48 weeks post D1	Immune responses HIV reservoir dynamics	Vaccines did not affect the size of the HIV reservoir in most PWH, except those with low-level viremia; vaccines were associated with increased viral blips
Qu, M. et al. (60) China	N = 38 PWH Vaccination -25 with BBIBP-CorV booster (homologous group) -13 with ZF2001 booster (heterologous group) Age BBIBP-CorV: 39 (IQR 30-49) ZF2001: 41 (IQR 37-56) Sex BBIBP-CorV: 24 (96.0%) male 1 (4.0%) female ZF2001: 12 (92.3%) male 1 (7.6%) female CD4^+^ T cell count BBIBP-CorV: 377 (IQR 278, 588) ZF2001: 426 (IQR 305, 580)	Inclusion criteria: -HIV diagnosis -Aged 18–60 years -Receiving ART -Plasma HIV RNA <20 copies/mL -No history of SARS-CoV-2 infection Exclusion criteria: -History of anaphylactic response to vaccine components -Current opportunistic infection -HBV, HCV, or influenza virus infection -Tumor and autoimmune diseases	BBIBP-CorV by Sinopharm (2 doses with a 28-day interval) BBIBP-CorV or XF2001 by ZIfivax (third dose at a 6-month interval with the second dose)	HIV-1 DNA – SUBPIO HIV Quantitative Detection Kit HIV RNA - SUBPIO HIV Quantitative Detection Kit	Expression of markers of T-cell phenotype and activation – flow cytometry with PBMCs based on the expression of CD45RA, CD27, and CCR7	Samples collected 10–13 months after 3^rd^ dose of vaccination, and 13–16 months after a breakthrough infection with SARS-CoV-2	HIV reservoirs T-cell immune recovery	ZF2001 group (heterologous booster dose): - higher CA-RNA levels after breakthrough infection - no significant differences in the level of HIV DNA -no correlation between CD4 counts and HIV DNA levels before and after breakthrough infection - baseline CD4 counts negatively associated with HIV CA-RNA post-breakthrough infection BBIBP-CorV group (homologous booster dose): - no significant differences in the level of HIV DNA - Higher HIV DNA levels pre-infections were strongly negatively correlated with lower baseline CD4^+^ T cell counts, rather than HIV CA-RNA Increased HIV reservoirs in PWH after breakthrough infection, especially in heterologous booster individuals
Rai, M. et al. (61) USA	9 PWH who received homologous booster -6 received Moderna -3 received Pfizer/BioNTech BNT162b2 vaccines Sex 8 male 1 female Race 6 Hispanic 1 White 1 Black 1 Mixed	Recruitment period: November 2021 to January 2022 Inclusion criteria: -HIV diagnosis -2-dose series of mRNA-based SARS-CoV-2 vaccines Exclusion criteria: Not specified	Moderna or Pfizer-BioNTech mRNA-based SARS-CoV-2 vaccines	Total HIV DNA Intact HIV proviral DNA – IPDA CA HIV RNA – RT-dPCR	S-specific Ig– ProcartaPlex Human Coronavirus Ig Total Panel 11-Plex assay and xMAP INTELLIFLEX Immune parameters – flow cytometry Frequencies of polyfunctional (IFNγ^+^, TNFα^+^, MIP1β^+^) HIV Gag-specific CD8^+^ T-cells – intracellular cytokines S-specific CD4^+^ T-cells– AIM assay	2–4 weeks before and 10–14 days post booster vaccination	Immune parameters, antibody responses, and HIV reservoir dynamics	No significant differences in the levels of total HIV DNA, intact HIV DNA and cell-associated HIV RNA in CD4^+^ T cells
Tuttle, D. et al. (62) USA	n = 139 -45 men with HIV (MWH) -94 men without HIV (MWOH) Median Age MWH: 67 MWOH: 62 Sex 139 (100%) male CD4^+^ T cell count Most MWH: 253–1888 cells/uL	Inclusion criteria Men with and without HIV-1 enrolled in the Pittsburgh Clinical Research Site of the MACS/WIHS Combined Cohort Study	2 doses of Moderna or Pfizer-BioNTech mRNA-based SARS-CoV-2 vaccines	HIV-1 residual plasma RNA - Integrase Single-Copy Assay (iSCA) HIV proviral DNA in CD4^+^ T cells - IPDA	S- and N-specific Ig G (for SARS-CoV-2 and endemic coronaviruses, C3 and C3a, and HIV-1 Nef – ELISA RBD-specific IgG and neutralizing antibodies against 3 SARS-CoV-2 variants – Meso Scale Discovery (MSD) and Plaque Reduction Neutralization Testing (PRNT)	Median time between vaccination and sample collection in days: MWH: 104 (12-249) MWOH: 101 (4-253)	SARS-CoV-2 vaccine immune responses in men with HIV-1	COVID-19 mRNA immunization reduced levels of HIV-1 RNA in the plasma (viremia) but did not alter the proviral HIV-1 DNA reservoir in CD4^+^ T cells.
Li, J. et al. (63) China	N=25 PWH Median age 34 (IQR 28.5-40.0) Sex: 23 (92%) male CD4 count: 461.9 (360.1–565.7) cells/uL	Recruitment period: March 2021 to February 2022. Inclusion: (1) No history of SARS-CoV-2 infection nor history of exposure to people diagnosed with COVID-19. (2) Willing to receive inactivated anti-SARS-CoV-2 vaccines. Exclusion: (1) pregnant, lactating or having planned pregnancy. (2) history of allergic reaction to vaccine ingredients or additives. (3) Participants with severe or active infectious disease within last month. (4) treated with immunosuppressive drugs. (5) had been vaccinated with another vaccine within 28 days before study, or are scheduled to be vaccinated during the study period.	CoronaVac (SinoVac, China) and Covilo (Sinopharm, China) (inactivated vaccines)	Total HIV-1 DNA Quantitative PCR Kit (SUPBIO)	Chemiluminescent immunoassay (CLIA) using SARS-CoV-2 kits (2019-nCoV IgG antibody detection kit; Bioscience Diagnostics)	The baseline visit, 20 days (IQR: 19 − 21) after first vaccine dose, 27 days (IQR: 26 − 28) after the second dose, 187 days (IQR: 180 − 192) after second dose and 14 days (IQR: 13 − 15) after the third dose.	pVL and HIV-1 total DNA	Receiving inactivated COVID-19 vaccine didn’t significantly affect pVL. HIV-1 total DNA copies had a downward trend after vaccination. Duration of infection and IgG titer might be correlated with HIV-1 total DNA copies after vaccination.
Song, B (64). China	53 PLWH on ART were vaccinated against COVID-19 with two doses of inactivated vaccine, with 35 receiving an inactivated vaccine (IN group) and 18 receiving a recombinant protein (CHO cells) vaccine as the third dose. Sex: 50 (%) male CD4: CD4^+^ T-cell counts (from 438 ± 45 cells/μL to 557 ± 48 cells/μL in CHO group, from 448 ± 34 cells/μL to 572 ± 39 cells/μL in IN group)	Recruitment Period: September 2021 to December 2021.	35 received a homologous booster dose, and 18 received a heterologous booster dose	Levels of cell-associated CA HIV DNA and CA HIV RNA- SUPBIO (Guangzhou, China) Viremia: quantified with the COBAS AmpliPrep/TaqMan real-time RT-PCR (Roche, CA, USA)	Immune measures/markers CD4^+^ and CD8^+^ T cell counts	Cross-sectional study 0, 2, and 8 months after the first vaccination	CA HIV RNA	CA HIV RNA levels differed significantly between the groups at month 8, with a more pronounced decrease in the IN group compared to the CHO group. The trends in HIV DNA were relatively similar between the two groups. CA HIV DNA initially decreased and then increased. Furthermore, while CA HIV RNA levels decreased in both groups, the reduction was more pronounced in the IN group. SARS-CoV-2 booster vaccine is safe for PLWH on ART, although it may affect HIV reservoirs and CD8^+^ T cell counts.
Kierii O et al (65) Sweden	N=37; 41% female; 22 IR (CD4≥350), 15 NIR (CD4<350); Median age 54 (33, 66); Ethnicity heterogeneous;	Recruitment Period: February 2021 to October 2021; Inclusion: eligibility for SARS-CoV-2 vaccination; Exclusion Criteria: Prior SARS-CoV-2 infection	BNT162b2 and mRNA-1273	Total, intact and defective (3’ and 5’) HIV DNA copies per million resting CD4+ T cells: IPDA with ddPCR (QX200, Bio-Rad); measured on day 0 and 3 months post 2^nd^ and 3rd doses	S- specific IgG (Elecsys Roch) at N-specific IgG (V-PLEX, MSD) S-specific CD4+ T cells by AIM assay	1st and 2nd doses BNT162b 21 days apart; 3rd dose BNT162b2 or mRNA-1273 given 9 month after 2nd dose	pVL; Intact, defective and total CA-HIV DNA; S- and N-specific IgG titers; S-specific CD4+ T cells	Vaccination does not significantly change HIV reservoir size regardless of immune status or immune reconstitution; Males tended to have higher reservoir size than females; Reservoir size correlated inversely with CD4+ T cell and nadir CD4+ T cell counts, and CD4/CD8 T cell ratio

*All studies examined CD4^+^ and CD8^+^ T cell frequencies as part of the standard of care.

AIM, Activation-induced marker; ART, Antiretroviral Therapy C3, Complement component 3; CA-HIV DNA, Cell-associated HIV DNA; CA-HIV RNA, Cell-associated HIV RNA; CHO, Chinese Hamster ovary cells; ddPCR, Droplet digital PCR; IPDA, Intact Proviral DNA Assay; IQR, Interquartile range; IR, Immune responders; LNTP, Long-term non-progressors; NIR, Non immune responders; PWH, People with HIV; SHCS, Swiss HIV Cohort Study; TILDA, Tat/Rev-induced.

## 5 HIV reservoir dynamics in PWH vaccinated for SARS-CoV-2

Most studies found no evidence that SARS-CoV-2 mRNA vaccination caused significant or long-lasting HIV reservoir perturbations. Duncan et al. (56) examined the effects of a two-dose mRNA vaccine on HIV viremia and reservoir markers in PWH, and reported no significant changes in the amount of intact HIV DNA, 5’-defective, 3’-defective and total proviral DNA copies per million CD4^+^ T-cells when comparing pre-vaccination, post-first vaccine dose and post-second vaccine dose time points (56). On a broader scale, the authors also compared the effect of mass vaccination for SARS-CoV-2 on the proportion of PWH with detectable viremia using the British Columbia Centre for Excellence in HIV/AIDS database, including all 290,401 plasma viral load (pVL) measures performed between 2012 and 2022. The comparison did not reveal evidence linking the mRNA vaccination to increases in the frequency of PWH on ART with detectable pVL in the province. Indeed, the proportion of detectable viral load among PWH in the province was identical in 2019 and 2022, at 18.5%, despite the mass rollout of anti-SARS-CoV-2 vaccinations in the province (56). This study therefore confirmed no significant perturbations in viremia and reservoir makers following the vaccination, both at the cohort and population levels. Another study in PWH, including 45 men and 94 women, in which the median time between vaccination and sample collection was 104 days (IQR 12-249), did not observe significant changes in the levels of HIV genomes (total, intact or defective) in CD4^+^ T cells (66). In fact, the authors noted a significant decrease in plasma viremia when measured by an assay that detects a single copy of the viral RNA/ml of plasma (67). Several studies, including a meta-analysis of 54 studies, found only a minor impact of two doses of anti-COVID-19 mRNA vaccines on HIV RNA in plasma examined at different time points post-vaccination (68–71).

At the cellular level, the SARS-CoV-2 mRNA vaccine was shown to induce a modest and transient transcriptional activation of latent HIV proviruses in PBMCs from PWH on ART with minimal changes in global T-cell activation (23). The vaccination, nevertheless, stimulated HIV-Nef-specific granzyme B^+^ CD8^+^ T-cell responses in vaccinated individuals. The authors suggested that by activating HIV transcription/replication, the vaccination exposed latently infected cells to Nef-specific CD8^+^ T cells (23). Interestingly, in this study, each of the T-cell response metrics that showed significant increases following the first vaccine dose strongly correlated with a decrease in cell-associated HIV RNA, a dynamic biomarker of HIV persistence (23, 72). The study (23) also reported an increase in cell-associated HIV RNA but not in plasma viremia. Thus, echoing the results on the two-dose vaccination regimen, the COVID-19 mRNA booster vaccine also does not appear to expand the HIV reservoir nor create an immunologic environment facilitating HIV reactivation in PWH receiving ART. Overall, these findings show that mRNA vaccination may reactivate latent HIV and kill HIV-producing cells by stimulating virus-specific CD8^+^ T cell responses, and thus may be helpful in HIV cure strategies such as those involving “shock & kill” (73–75). Another study also highlighted the safety of the booster dose, and showed that the dose did not induce significant differences in the levels of total HIV DNA, intact proviral DNA or cell-associated HIV RNA in CD4^+^ T cells (61). Given that new variants of SARS-CoV-2 are emerging, booster vaccinations remain effective at reducing the risk of severe COVID-19 disease, and are thus crucial for high-risk populations, including PWH (76). As the literature does not demonstrate a risk of reservoir disturbance in PWH, guidelines on COVID-19 boosters should integrate these tailored findings to ensure adequate uptake in the population.

Very few studies have specifically investigated COVID-19 vaccination outcomes in distinct subgroups of PWH. Given that older individuals may experience diminished immune responses due to inflammaging and immunosenescence, one study examined the impact of vaccination in PWH aged >55 years with age-matched HIV negative controls (59). This focus is particularly relevant in the context of the aging demographics of the HIV population, with the advent of ART and the possible expansion of HIV reservoirs with age (77). The study found that after two doses, PWH showed suboptimal immune response compared with HIV negative controls. Overall, the size of the HIV reservoir remained unchanged following COVID-19 vaccination in PWH with a suppressed viral load but increased in PWH with pre-existing low-level viremia (59). In addition, the vaccination was associated with increased transient episodes of detectable HIV RNA (59). This study was conducted in Ontario, Canada, where participants had received different vaccination regimens, comprising mRNA- and chimpanzee adenovirus vector-based vaccines. Compared to HIV-negative participants, PWH demonstrated weaker and less durable salivary IgA responses following vaccination, suggesting reduced mucosal protection against SARS-CoV-2 infection. Additionally, compared to age-matched HIV-negative participants, PWH had lower neutralizing antibody titers after 2 vaccine doses. Importantly, the immune responses were enhanced by a third dose, emphasizing its importance in reinforcing protection against SARS-CoV-2 infection in aged PWH. Interestingly, S-specific T cell responses in immune-responder PWH were even higher than those of HIV-negative controls (59). The authors noted transient increases in CD4^+^ T cells; however, they did not observe increases in Nef- or Gag-specific T cells after two or three vaccine doses as reported by Stevenson et al (23). Another study (65) investigated the impact of 3 doses of mRNA-based SARS-CoV-2 vaccines, 2–3 months after the 3^rd^ dose, on the reservoir size in immune responders (<350 CD4^+^ T cells/µl) versus non-immune responders (≥ 350 CD4^+^ T cells/µl), as well as in male versus female PWH. The authors found no significant effect of the vaccination on the reservoir size. They also reported no differences in the reservoir size between responders and non-responders; however, the size correlated inversely with CD4^+^ T cell counts and CD4^+^ T cell/CD8+ T cell ratios (65). Furthermore, they found that the reservoir size tended to be higher in males than in females, although the difference was not statistically significant (65).

Another sub-analysis of COVID-19 vaccination outcomes in PWH is a case report of a 65-year-old HIV-1 elite controller woman, who experienced viral rebound after SARS-CoV-2 vaccination (57). Ultimately, the post-vaccination rise in viremia in this case was controlled without ART intervention (57). HIV-RNA undetectability was achieved 10 months after the third dose of the vaccine, suggesting that the viral rebound was, in fact, transient (57). To the best of our knowledge, this is the only elite controller who, upon COVID-19 vaccination, experienced an increase in plasma viremia.

Data from regions where inactivated virus vaccines were used are limited but provide additional context. For example, Peng et al. reported that CoronaVac induced transient increases in cell-associated HIV RNA and HIV DNA at 2 and 12 weeks post-second vaccine dose (58). However, follow-up 2–3 weeks post-third dose (administered 12 weeks post-second dose), the viral load remained under the limit of detection (20 copies/mL). More recently, Li et al. reported no effect on pVL after one, two and three doses of inactivated vaccines (CoronaVac and BBIBP-CorV) (63). However, they noted a downward trend in total HIV DNA in CD4^+^ T cells in vaccinated PWH irrespective of their baseline CD4^+^ T cell counts.

A higher incidence of breakthrough infections in PWH as compared with that in PWOH has been reported even after a booster dose (78–80). In this context, Qu et al. investigated the impact of homologous and heterologous booster doses on the viral reservoir in PWH who had been previously vaccinated with two doses of inactivated BBIBP-CorV and experienced a breakthrough infection (60). The homologous booster vaccine was BBIBP-CorV, and the heterologous booster dose was ZF2001, a recombinant protein subunit vaccine (60). Both homologous and heterologous booster vaccinations seemed to actually curb the expansion of the HIV reservoir, particularly the increase of HIV DNA, in individuals with breakthrough infections (23, 59). The heterologous booster, however, induced stronger immune activation and smaller reduction in reservoir compared to the homologous one. These findings contrast with those from an earlier study by Peng et al. with inactivated vaccines (58). However, Qu et al. measured total HIV DNA, which may not accurately reflect the size of the HIV reservoir, as it may also include unintegrated HIV DNA, which decays naturally over time. The measure also does not distinguish between intact and defective proviruses (40, 60). Song et al. investigated the impact of anti-SARS-CoV-2 vaccination in PWH, who were administered two doses of an inactivated vaccine and received either a booster dose with homologous inactivated vaccine or with a heterologous vaccine (recombinant S protein produced in CHO cells) (64). They followed the participants for 8 and 13 months post-first dose. Both vaccine regimens significantly increased CD4^+^ and CD8^+^ T cell counts in the vaccinees compared with the pre-vaccination baseline counts. The CA-HIV DNA decreased at month 2 but increased significantly at month 8 post-first dose of the vaccination. In contrast, the CA-HIV RNA levels continued to decrease at both these time points. These results suggest that inactivated vaccination may increase specific markers of HIV persistence. Collectively, these studies suggest a minor impact of repeated anti-SARS-CoV-2 vaccination, if any, on the HIV reservoir in PWH.

## 6 Potential factors contributing to similarities/differences in study results

### 6.1 Vaccine type

The predominant use of mRNA vaccines in North America and Europe contrasts with the reliance on adenovirus-vectored or inactivated vaccines in other regions of the World, such as China, India and South America. Despite their distinct mechanisms of action, the literature supports the overall safety of COVID-19 vaccination in PWH, with no viral rebound or durable increase of HIV reservoir markers reported in current studies. Nonetheless, the vaccine platform may partially account for transient increases in HIV RNA or DNA observed in some studies of inactivated vaccines (60).

### 6.2 Study design and outcome measures

Differences in methodological approaches also complicate comparisons between studies. Some studies used highly sensitive assays to measure intact proviruses, such as the IPDA, which can precisely measure the absolute number of HIV genomes within a sample without the need for a standard curve and can distinguish between defective and intact proviruses, while others relied on relatively less precise methods for HIV DNA quantification, which do not differentiate between intact and defective proviruses (81). Peng et al. reported transient increases in total HIV DNA post-vaccination with an inactivated virus, but without intact proviral analysis, the clinical relevance of these findings remains uncertain (58).

Different studies collected samples at different post-vaccination time points, which limits valid comparisons between them. Indeed, studies assessing participants more than 2 weeks post-vaccination, such as the one by Duncan et al., may have missed earlier viremia events, which usually occur within 2 weeks following vaccination (56). Conversely, the study by Peng et al. that identified a transient increase in HIV DNA post-vaccination and cell-associated HIV RNA might have only been detected due to the timing of the follow-up visit (58). Furthermore, SARS-CoV-2-mediated immune activation, immunogenicity and inflammatory response may vary depending on whether a homologous or heterologous vaccine is used as a booster. Heterologous booster doses induce a more potent immune response and are associated with stronger effects on HIV persistence markers (60).

Studies investigating HIV reservoirs evaluate reservoir dynamics based on laboratory measures, such as CA HIV DNA and RNA, rather than clinical outcomes. Of note, it is unclear whether changes in HIV reservoir size are associated with any clinical outcome.

### 6.3 Participant demographics

All studies included in this review predominantly enrolled male participants. In addition, most participants in North American cohorts were white, whereas Chinese studies naturally included East Asian participants. As such, this relative homogeneity enhances internal consistency amongst the current studies. The median age of participants was also rather consistent across the studies, with most enrolling participants with a median age in their 40s, and two in their 60s. In the context of infections with SARS-CoV-2, age is important to consider, as it is an independent risk factor for severe outcomes (82). Likewise, HIV reservoirs expand with age, in part due to sex-specific mechanisms, such as hormonal fluctuations (77). These underlying influences, however, have not been thoroughly explored in existing studies on anti-SARS-CoV-2 vaccination in PWH. In the same vein, the risk of worse COVID-19 outcomes increases with the number of comorbidities (26). Many studies in this review excluded PWH with chronic or acute medical conditions, resulting in a bias in the enrolment of relatively healthy participants. Although these stringent criteria improve comparability between studies, they limit the applicability of findings to the broader population of PWH.

### 6.4 ART status and baseline viremia

Most of the studies were conducted in virally suppressed individuals on ART, allowing their direct comparison. However, special cases suggest that vaccine-induced immune activation may vary in unique subgroups of PWH. Indeed, the elite controller who experienced a transient viral rebound after vaccination and older PWH with pre-existing lower-level viremia were more likely to experience viral blips post-vaccination, highlighting that clinical phenotypes may modify anti-SARS-CoV-2 vaccination outcomes (57, 59).

### 6.5 Sample size and study scope

Most studies on SARS-CoV-2 vaccine outcomes in PWH relied on small sample sizes and lacked HIV-negative control groups, which may have caused variability in the interpretation of results.

## 7 Strengths

Overall, the literature is consistent across multiple cohorts and methodological approaches in indicating that SARS-CoV-2 vaccination does not significantly perturb HIV reservoirs or plasma viremia in PWH. Large-scale analyses, such as those from the British Columbia Centre for Excellence in HIV/AIDS, provide reassurance at both the cohort and population levels by showing that neither an increase in detectable viremia nor a durable disturbance in viral reservoir occurs following vaccination (56). Furthermore, a few studies extended their analyses beyond the initial two-dose regimen to evaluate the effects of booster vaccination (59, 60). These investigations further confirmed the safety of booster administration in PWH with respect to viral rebound and reservoir size. Another methodological strength of the existing studies is the repeated sampling of participants in nearly all studies. By collecting samples at multiple time points, often before vaccination and after each dose, researchers were able to track temporal changes in reservoir dynamics. This approach allowed for the identification of transient fluctuations, such as short-lived viral blips, while distinguishing them from more durable virologic or immunologic effects. By incorporating booster data, which demonstrate the tolerability of COVID-19 vaccination beyond the two-dose regimen, the literature offers findings that are essential to vaccination campaigns and evolving public health recommendations.

## 8 Weaknesses

Several limitations should be considered in interpreting these findings. As mentioned, most studies to date involve relatively small sample sizes, restricting the ability to detect subtle or delayed effects on reservoir size and stability. Similarly, small cohort sizes inherently limit participant diversity, which can, by extension, diminish the significance and applicability of observed patterns. In the existing literature, the predominantly homogenous makeup of participants constrains generalizability to women, racial minorities, and other underrepresented groups of PWH who may experience distinct vaccine responses or reservoir perturbation. In fact, sex-based differences in HIV reservoir size, immune activation, and response to ART are well-documented in the literature, but not in the context of anti-SARS-CoV-2 vaccination, leading to an important knowledge gap (83). Likewise, outcomes in individuals with unsuppressed viremia, advanced immunosuppression, or incomplete ART adherence remain underexplored. A lack of their participation in these studies may be due to stringent inclusion criteria; indeed, a few studies discussed in this review specifically excluded PWH with acute or chronic medical conditions or pVL >200 copies/ml (23, 58). Finally, an important factor that has also been ignored in these studies is the duration of ART in participating PWH.

As previously alluded to, methodological heterogeneity in HIV reservoir measurements and in the time points studied adds a caveat to the interpretation of certain results (40, 84, 85). The lack of long-term monitoring post-vaccination beyond several months highlights a gap in longitudinal data. Most studies in this review evaluated participants as early as 2 weeks post-vaccination, and may have missed viral blips occurring at earlier time points. Finally, the limited amount of available data is derived from different types of SARS-CoV-2 vaccines, namely mRNA-based, subunit, vectored and whole inactivated virus. While PWH in high-income countries received mRNA vaccines, fewer studies have examined adenovirus vector-based or inactivated vaccines, which remain widely used globally. The discrepancies in findings among studies may therefore be due to the use of different vaccine types, as evidenced by the association between inactivated influenza vaccines and an elevated HIV proviral burden (12).

Future research should prioritize longitudinal, multi-cohort studies with standardized reservoir measurement assays to clarify the long-term impact of repeated vaccination, including boosters, across diverse vaccine platforms. Furthermore, studies stratifying participants by clinical and demographic variables, including sex, age, duration of AT, comorbidities and viral load, will be essential to determine whether certain PWH subgroups are more susceptible to vaccine-induced reservoir perturbations. Ultimately, understanding how vaccination influences HIV persistence could simultaneously address the higher rate of vaccine hesitancy among PWH and provide insights for HIV cure research, particularly regarding latency reversal and immune modulation strategies.

## 9 Conclusions

Only a few studies have investigated the impact of repeated anti-SARS-CoV-2 vaccination on HIV reservoir dynamics. Currently, the vaccination and booster doses appear to be safe in PWH and do not significantly perturb the HIV reservoir in PWH who are on ART, have effectively suppressed viral replication and have undetectable viremia. These vaccinations, however, should be closely monitored in PWH who are not receiving ART (such as elite controllers), are non-compliant with ART and have detectable viremia, are immune non-responders or have a higher burden of co-morbidities. Current evidence suggests that SARS-CoV-2 vaccines do not compromise long-term virologic control or reservoir stability in PWH; however, they should be continuously monitored in larger studies across diverse vaccine platforms, cohorts and genders.
